# Glabellar Rhytids in Thyroid Eye Disease: Severity and Their Impact on Emotional Perception

**DOI:** 10.1111/jocd.71243

**Published:** 2026-10-21

**Authors:** Mannat Kaur, Noemi D. Rivera, Sugi P. Lazarus, Sara T. Wester

**Affiliations:** ^1^ Bascom Palmer Eye Institute, Department of Ophthalmology University of Miami Miller School of Medicine Miami Florida USA

**Keywords:** botulinum toxin, emotional perception, facial perception, glabellar rhytids, psychosocial burden, thyroid eye disease

## Abstract

**Purpose:**

To quantify glabellar rhytid severity in patients with Thyroid Eye Disease (TED) compared to age‐and sex‐matched controls and to evaluate its impact on external emotional perception.

**Methods:**

A matched case–control study was performed comparing patients with TED of at least 6 months duration to controls (healthy individuals with no history of TED). Standardized photographs were cropped to the glabellar region. Controls were matched to cases by sex and age within five‐year intervals. Images were independently evaluated by masked assessors. Glabellar rhytid severity was graded using a validated 4‐point glabellar‐line severity scale. Perceived emotion expression was assessed across 7 categories: neutral, anger, disgust, sadness, fear, happiness, and surprise.

**Results:**

A total of 90 patients (45 TED and 45 controls) were analyzed by 9 independent assessors. Patients with TED demonstrated significantly greater glabellar rhytid severity than controls (mean GLS score, 2.55 vs. 2.23 respectively; paired *t*‐test, *p* = 0.013). A generalized linear mixed model accounting for reviewer variability and matched‐pair design demonstrated that TED images had 47% higher odds of receiving negative emotion rating than control images (OR = 1.47, 95% CI = 1.06–2.02; *p* = 0.019). Compared with controls, TED images were frequently perceived as expressing anger and disgust and less frequently perceived as neutral. Glabellar rhytid severity correlated positively with proptosis severity.

**Conclusions:**

Patients with TED exhibit significantly more severe glabellar rhytids than age‐and sex‐matched controls and had more negative attribution of negative emotion. These changes may potentially contribute to the psychosocial burden and social stigma associated with TED. Early targeted interventions, including botulinum toxin, should be considered in appropriately selected patients.

## 1 Introduction

Thyroid eye disease (TED) is a complex, chronic autoimmune disease characterized by expansion and remodeling of orbital and periorbital tissues [1, 2]. The resulting structural changes give rise to hallmark clinical features, such as proptosis and eyelid retraction, which significantly alter ocular surface physiology by increasing corneal exposure. This exacerbates dryness and leads to photophobia and ocular surface discomfort [3, 4, 5]. Eyelid retraction results from a combination of inflammation, fibrosis, and restriction of vertical rectus muscles, leading to an increased palpebral fissure height [6]. Concurrently, altered ocular surface expression of proinflammatory cytokines perpetuates ocular surface disease [7]. In response to this increased exposure and surface irritation, patients often develop compensatory overaction of the accessory protractor muscles, primarily the procerus and corrugator supercilii, in an attempt to reduce the vertical palpebral fissure and alleviate symptoms [8, 9, 10]. Many patients also engage these muscles in an attempt to mask the disfiguring effects of a widened stare and strabismus [9]. Persistent contraction of these muscles can result in prominent glabellar rhytids, often presenting as deep horizontal and oblique lines across the glabellar region [10]. These rhytids are distinct from fine, age‐related facial wrinkles caused by dermal collagen age changes [10, 11].

Despite being a frequently observed clinical finding, glabellar rhytids in TED remain underrecognized and poorly studied. In a retrospective study by Saks et al., 82.5% of patients with TED demonstrated glabellar rhytids, with severity increasing in parallel with worsening disease features [9]. In combination with proptosis and eyelid retraction, these rhytids contribute to the characteristic “TED facies” which carries both functional and cosmetic consequences. The eyes are central to emotional expression and influence how an individual is perceived by others [12]. Proptosis, lid retraction, and conjunctival injection are visual cues that are associated with anger, fear, and surprise [12, 13]. These changes can be psychologically distressing and may negatively influence social interactions, with many patients describing their appearance as an “angry scowl” [10, 12, 14, 15, 16].

Importantly, even after successful restoration of visual function, many TED patients remain dissatisfied with their appearance. Studies suggest that up to 60.5% of patients feel their eyes have not returned to their pre‐disease appearance, underscoring the significant aesthetic burden of TED and its impact on quality of life [17, 18]. Farid et.al further reported that the emotional distress in TED patients is often driven more by disfigurement rather than by functional deficits [18].

Despite potentially significant contributions of glabellar rhytids to aesthetics and social implications of facial changes in TED, they have not been quantitatively assessed relative to healthy age‐matched controls. Furthermore, no prior studies have evaluated how these features are perceived and whether they influence social impressions through emotional attribution. This retrospective case–control study aimed to characterize glabellar rhytids in patients with TED, quantify their severity relative to matched controls, and evaluate whether these changes influence emotional perception by independent observers. By examining both anatomical manifestations and their social consequences, this study expands the understanding of the disease burden of TED beyond traditional functional outcomes.

## 2 Materials and Methods

This study adhered to the Health Insurance Portability and Accountability Act as well as to the ethical principles outlined in the Declaration of Helsinki.

An electronic record search was performed to evaluate patients between July 1, 2024, and January 1, 2026. Patients with a clinical diagnosis of TED comprised the TED group and patients evaluated for functional dermatochalasis or ptosis without a history of TED served as controls. An initial search identified 320 patients. A manual chart review was conducted to confirm eligibility. Inclusion criteria consisted of patients ≥ 18 years of age and availability of standardized facial photographs obtained in a neutral facial expression. Exclusion criteria included prior cosmetic procedures (Botox injections, dermal fillers, forehead lift, brow lift, or laser resurfacing), TED duration of ≤ 6 months, history of major facial trauma, or neuromuscular disorders affecting facial musculature. Following application of inclusion and exclusion criteria, 168 patients were eligible. Propensity score matching was performed based on sex and age (5‐year age bins). This yielded 45 matched TED‐control pairs (90 total patients), which constituted the final study cohort. Manual chart review was conducted to collect demographic and clinical data at the time of photograph including ethnicity, smoking status, prior TED treatment, duration of TED, Clinical activity score (CAS), Hertel exophthalmometry, Gorman Diplopia score (GDS), BMI, and systemic thyroid status.

A survey was developed using Qualtrics (Qualtrics, Provo, UT, USA). Standardized facial photographs were cropped to only include the forehead and glabellar region (Figure 1A).

**FIGURE 1:**
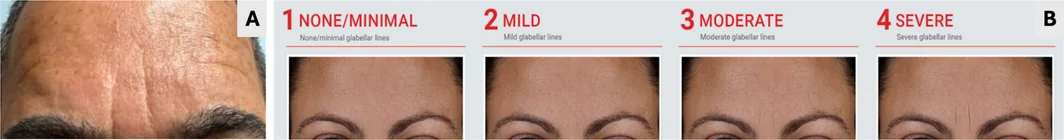
(A) Standardized facial photographs cropped to the forehead and glabellar region (B) Photo numeric scale of glabellar lines at rest [19].

Images were randomized using an online random list generator to eliminate systematic ordering bias before incorporation into the survey. The survey was distributed electronically to independent, non‐medical reviewers including assistants, undergraduate students, and graduate students. Each photograph was evaluated twice, once for glabellar line severity rating and second for emotional perception. Reviewers graded glabellar line severity using the validated 4‐point Glabellar Line Scale (GLS) (Ratings from 1 to 4 where 1 = None; 2 = Mild; 3 = Moderate and 4 = Severe) [19, 20, 21] and assessed perceived emotion from the following list: neutral, anger, disgust, sadness, fear, happiness, or surprise. Standardized instructions outlining the study objectives and the GLS scale, including representative images illustrating each severity category, were provided before image evaluation (Figure 1B). To capture first‐impression rhytid severity and emotional perception, each photograph was displayed for 5 s before automatically advancing to the response screen.

For statistical analysis, emotions were further classified into valence categories described by Baudouin et al. [22] Anger, disgust, sadness, and fear were categorized as negative‐valence emotions; happiness as positive‐valence; and surprise and neutral as non‐valenced emotions [22]. All statistical tests were performed using IBM SPSS statistics (IBM Corp., Armonk, NY, USA). Statistical methods included paired *t*‐test, intraclass coefficient (ICC) analysis, generalized linear mixed‐effects models (GLMMs), Fleiss' multirater kappa, Spearman's rank correlation analysis, Fisher's exact test, and the Mann–Whitney *U* test. A significance threshold of *p* < 0.05 was used for all tests.

## 3 Results

The study included 90 patients (45 TED and 45 age‐and sex‐matched controls). The mean age of the cohort was 61.96 ± 10.84 years (range, 35–86 years) and 70 patients (77.78%) were female. The cohort was predominantly Hispanic (59.3%) followed by White (29.1%), African American (7%) and Asian (4.7%). Compared with controls, the TED group included a greater proportion of African American patients (6 vs. 0 *p* = 0.01) and Asian patients (4 vs. 0; *p* = 0.048). Smoking status was similar between groups with 6.7% current smokers, 29.2% former and 64% never smokers. Mean BMI was 29.21 kg/m^2^ in TED group and 27.51 kg/m^2^ in control group.

Among the TED group, mean disease duration at the time of photograph was 62.9 months. The mean CAS was 2.6 and mean Hertel exophthalmometry measurements were 22.75 mm for the right eye and 22.79 mm for the left eye. 14 of the 45 TED patients (31.1%) had received prior treatment for TED including corticosteroids (*n* = 8), decompression surgery (*n* = 3), teprotumumab (*n* = 3), orbital radiation therapy (*n* = 1), lid retraction surgery (*n* = 1) and tocilizumab (*n* = 1). Thyroid function status was hyperthyroid in 12 patients (26.7%), hypothyroid in 3 (6.7%), euthyroid in 29 (64.4%) and unknown in 1 (2.2%).

Nine independent reviewers evaluated all 90 photographs, generating 810 total ratings: 405 GLS ratings and 405 emotion ratings.

Patients with TED demonstrated significantly greater glabellar rhytid severity than controls (mean GLS score, 2.55 vs. 2.23 respectively; paired *t*‐test, *p* = 0.013; Figure 2). The Inter‐rater reliability was assessed using the ICC. The Single‐Measures ICC was 0.573 (95% CI, 0.486–0.662) indicating “moderate” agreement among individual reviewers. The Average‐Measures ICC was 0.924 (95% CI 0.895–0.946), demonstrating “excellent” agreement when ratings were averaged across reviewers. The overall distribution of GLS rating votes is summarized in Table 1. Across the total 405 GLS ratings observed in each group, TED images were more frequently graded as having “moderate” or “severe” glabellar rhytids than control images.

**FIGURE 2:**
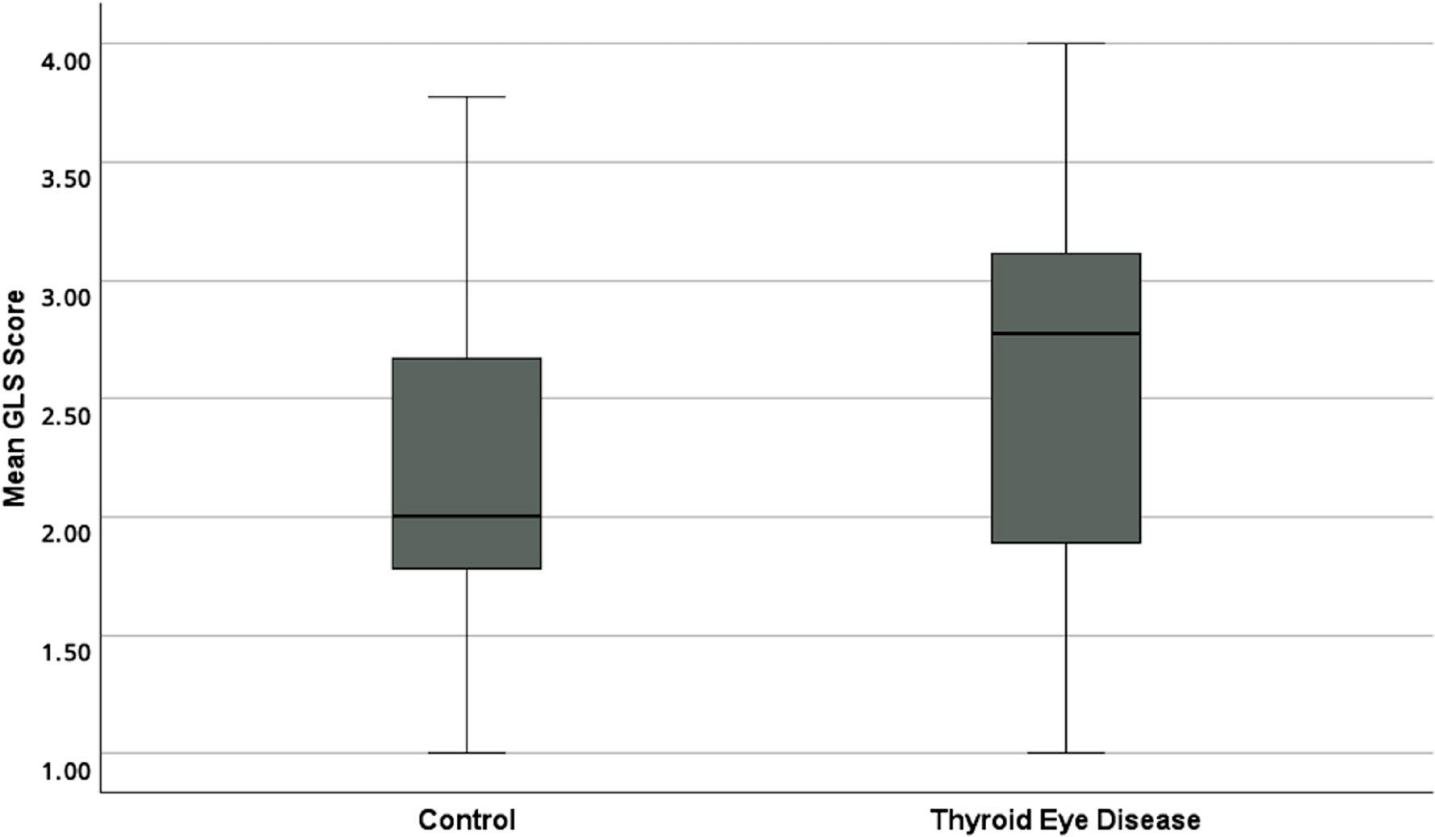
Box plot of glabellar line severity (GLS) score in Control and Thyroid Eye Disease groups. The central line represents the median, the box represents the interquartile range (IQR), and the whiskers indicate the observed range. GLS, Glabellar Line Severity; IQR, Interquartile range.

**TABLE 1:** Overall glabellar line severity ratings from all independent reviewers.

Glabellar line severity scale	Controls, *n* (%)	TED, *n* (%)
None	84 (20.49%)	68 (16.79%)
Mild	187 (46.17%)	136 (33.58%)
Moderate	90 (22.22%)	111 (27.4%)
Severe	44 (10.86%)	90 (22.22%)
Total	405	405

Abbreviations: *n*, total number; TED, thyroid eye disease.

A GLMM accounting for reviewer variability and the matched‐pair design demonstrated that TED images had significantly higher odds of receiving a negative emotion rating than matched control images (OR = 1.47, 95% CI = 1.06–2.02; *p* = 0.019; Figure 3). Inter‐rater agreement for emotion classification was assessed using Fleiss' multirater Kappa test. Overall agreement across emotion categories was “fair” (weighted *κ* 0.216). Agreement for the classification of negative emotion was “Moderate” (weighted *κ* 0.599). The overall distribution of emotion ratings is summarized in Table 2. Across the 405 emotion ratings, TED images demonstrated a higher proportion of “anger” and “disgust” ratings and a lower proportion of “neutral” ratings compared with the control ratings.

**FIGURE 3:**
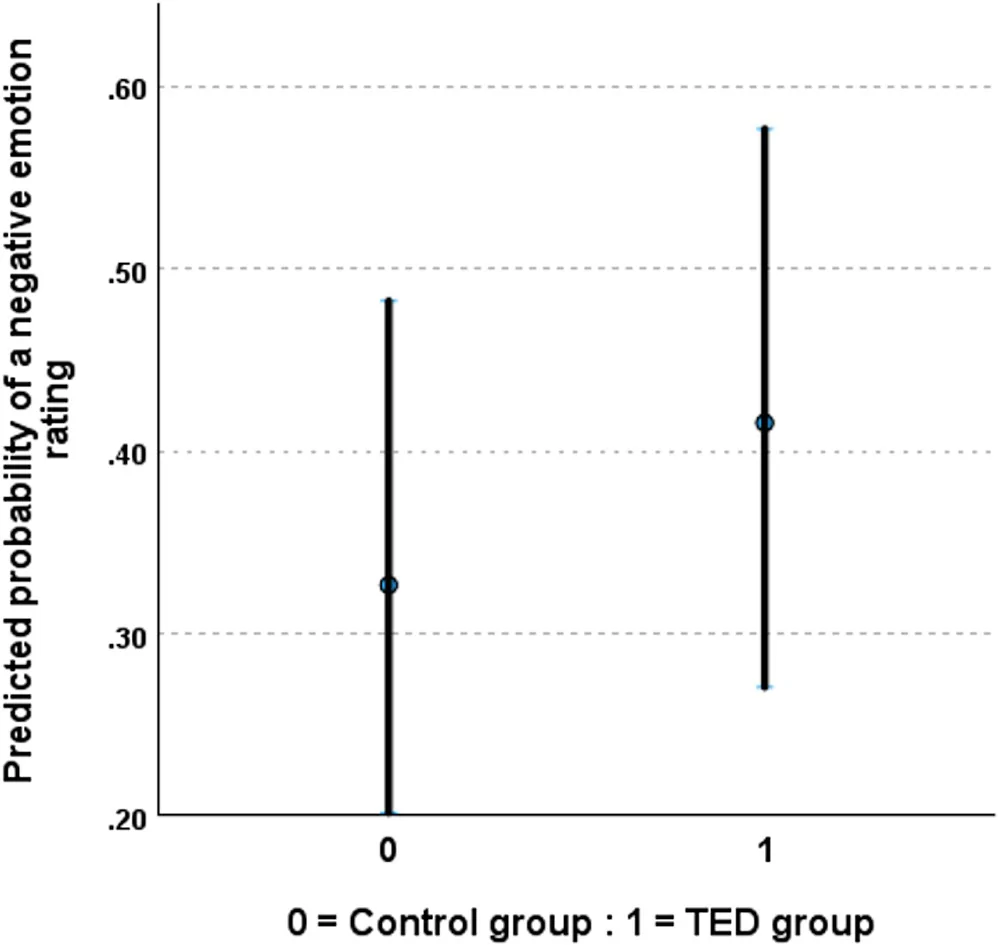
Adjusted predicted probabilities of negative emotion ratings for control and Thyroid Eye Disease (TED) images derived from a generalized linear mixed‐effects model accounting for reviewer variability and matched image pairs. Error bars represent 95% confidence intervals. The model demonstrated significantly higher odds of negative emotion ratings for TED images compared with control images (Odds Ratio = 1.47, 95% Confidence Interval = 1.06–2.02; *p* = 0.019). TED, Thyroid Eye Disease.

**TABLE 2:** Overall distribution of emotion rating votes from all independent reviewers.

Emotion rating	Controls, *n* (%)	TED, *n* (%)
Neutral	173 (42.71%)	135 (33.33%)
Anger	46 (11.36%)	80 (19.75%)
Disgust	24 (5.92%)	37 (9.14%)
Sad	57 (14.07%)	33 (8.15%)
Fear	22 (5.43%)	28 (6.91%)
Happy	43 (10.62%)	41 (10.12%)
Surprised	40 (9.88%)	51 (12.59%)
Total	405	405

Abbreviations: *n*, total number; TED, thyroid eye disease.

Within the TED cohort, Spearman's correlation analysis demonstrated significant positive correlations between GLS score and Hertel exophthalmometry measurements for both the right eye (*ρ =* 0.477, *p* = 0.001) and left eye (*ρ* = 0.317, *p =* 0.036). No significant correlation was observed between GLS ratings and CAS, GDS, or the duration of TED.

Additionally, no significant associations were identified between GLS scores and smoking status, BMI, or ethnicity in the TED cohort, control cohort, or the combined study population.

## 4 Discussion

TED produces characteristic orbital and periocular changes that profoundly alter facial appearance; however, the contribution of these changes to social perception and the associated psychosocial burden remains underrecognized. To our knowledge, this is the first study to quantitatively compare glabellar rhytid severity between TED patients and age‐and sex‐matched controls and examine its association with emotional perception by independent observers.

Patients with TED demonstrated significantly greater rhytid severity than matched controls (*p* = 0.013), with TED images more frequently graded as having “moderate” or “severe” glabellar rhytids than control images. (Figure 2; Table 1) Previous work by Saks et al. reported that more than 80% of patients with TED exhibited glabellar rhytids, with increasing severity paralleling worsening disease manifestations [9]. Our findings extend this observation by demonstrating that glabellar rhytids are significantly more pronounced than those observed in matched controls, suggesting that these changes exceed those expected from normal aging alone. Furthermore, the positive correlation between glabellar rhytid severity and Hertel exophthalmometry measurements supports an association between orbital disease severity and glabellar muscle overactivity. One possible explanation is that progressive proptosis, by increasing the palpebral fissure length and ocular surface exposure, may promote chronic compensatory activation of the corrugator supercilii and procerus muscles, potentially contributing to the permanent, deep glabellar furrowing [8, 9, 10].

Furthermore, TED images had 47% higher odds of being perceived as expressing negative emotion compared with matched controls (OR = 1.47, 95% CI = 1.06–2.02; *p* = 0.019; Figure 3). Specifically, TED images were more frequently classified as conveying “anger” and “disgust” and less frequently perceived as “neutral” than control images (Table 2). These findings suggest that characteristic facial changes associated with TED influence emotional perception during social interactions. Prior studies have shown that appearance‐related concerns frequently outweigh functional impairment in determining the quality of life among patients with TED, and that dissatisfaction with facial appearance often persists despite successful treatment of visual symptoms [17, 18]. Our findings extend this literature by suggesting that glabellar rhytids may represent a previously underrecognized contributor to this persistent psychosocial burden, as they may inadvertently convey negative emotional expressions to observers. This may partially contribute to the social stigma, impaired interpersonal interactions, and reduced quality of life commonly reported by patients with TED. Importantly, the photographs used in our study did not include the eyes and provided limited visualization of eyelids, therefore minimizing the potential contribution of lid‐retraction and conjunctival injection, the characteristic findings of TED, that are well recognized as visual cues associated with expressions of anger and fear. Recognizing glabellar changes as a component of the characteristic “TED facies” broadens the understanding of disease burden, and emphasizes the importance of incorporating facial appearance and its psychosocial consequences during comprehensive clinical assessments of patients with TED.

These observations also have important therapeutic implications. Botulinum toxin injections into the glabellar complex are an established treatment for glabellar rhytids and have been shown to improve appearance‐related satisfaction and self‐confidence [23]. In patients with TED, they may provide benefits beyond aesthetic improvement. By reducing deep glabellar furrowing, treatment may help mitigate unintended negative emotional attribution by observers, thereby improving social interactions and psychosocial well‐being. Our findings strengthen the rationale for earlier interventions in appropriately selected patients, and botulinum toxin may warrant consideration as a part of a comprehensive rehabilitative approach to TED rather than being viewed solely as a cosmetic intervention. Although this will require further prospective evaluation, these broader therapeutic benefits may warrant reconsideration of current reimbursement policies and insurance coverage for appropriately selected patients with TED.

Several limitations merit consideration. This was a single center study with a relatively modest sample size, which may limit the generalizability of the findings. Although photographs were obtained under standardized conditions, static two‐dimensional images cannot fully capture the dynamic facial expression encountered during normal social interaction. While overall inter‐rater agreement for emotion classification was fair, agreement for identifying negative emotions was substantially stronger. Importantly, GLMM accounted for this variability among raters, supporting the robustness of the primary outcome. Future studies should investigate the longitudinal evolution of glabellar rhytids throughout the course of TED and determine whether medical or surgical treatments alter rhytid severity, emotional perception, and patient‐reported outcomes.

## 5 Conclusion

Glabellar rhytids represent a previously underrecognized facial manifestation of TED, associated not only with disease severity but also with an increased likelihood of negative emotional attribution by observers. These findings contextualize the social stigma experienced by many patients with TED and underscore the importance of a comprehensive approach to disease management that addresses both the functional and psychosocial consequences of the disease.

## Funding

M.K., S.P.L., S.T.W. were supported by an unrestricted grant to the Bascom Palmer Eye Institute from Research to Prevent Blindness. (GR004596‐1).

## Ethics Statement

The study was approved by the Institutional Review Board at the University of Miami.

## Conflicts of Interest

S.T.W. is a consultant/advisor for Amgen/Horizon therapeutics, Immunovant, Argenx, Sling, Kriya; Contracted research with Amgen//Horizon Therapeutics, Immunovant, Sling, Lassen, Tourmaline, Argenx. The other authors declare no conflicts of interest.

## Data Availability

The data that support the findings of this study are available from the corresponding author upon reasonable request.
